# Shared reading as an intervention to improve health and well-being in adults: a scoping review

**DOI:** 10.3389/fpsyg.2025.1484839

**Published:** 2025-03-28

**Authors:** Kajsa Järvholm, Anders Ohlsson, Katarina Bernhardsson, Anna W. Gustafsson, Per Johnsson, Martin Malmström, Jonatan Wistrand, Torbjörn Forslid

**Affiliations:** ^1^Department of Psychology, Lund University, Lund, Sweden; ^2^Centre for Languages and Literature, Lund University, Lund, Sweden; ^3^Birgit Rausing Centre for Medical Humanities, Lund University, Lund, Sweden; ^4^Department of Culture, Languages and Media, Malmö University, Malmö, Sweden; ^5^Department of Clinical Sciences, Medical Faculty, Lund University, Lund, Sweden

**Keywords:** literature, culture and health, quality of life, depression, group intervention, community

## Abstract

**Background:**

Shared reading (SR) is a group reading concept consisting of weekly meetings led by a trained facilitator where literary fiction and poetry is read aloud and discussed. SR requires no previous knowledge or homework and has been tried out as a health intervention to different vulnerable populations.

**Objectives:**

The present study undertook a scoping review of research evaluating SR as an intervention to improve health and/or well-being in adults. The aim was to map the existing knowledge, identify research gaps, and suggest how these gaps can be addressed in future research.

**Method:**

We followed the PRISMA guidelines for scoping reviews. Online data bases were searched for publications on SR published between Jan 1, 2005, and Dec 31, 2024. Studies were eligible if they used SR as an intervention for adults, reported outcomes for health and/or well-being, and were published in Danish, English, Norwegian, or Swedish in peer-reviewed journals. Quantitative, qualitative, and mixed-methods studies could be included.

**Results:**

The search returned 179 records. We identified 15 studies, all written in English, that met the inclusion criteria. The studies were conducted in UK (67%; *n* = 10) and in the Scandinavian countries (33%; *n* = 5). Participants were mostly vulnerable populations such as people with dementia, mental illness, or chronic pain. The number of included participants varied between 4 and 61. Quantitative data were reported in 47% (*n* = 7) of the studies, showing improvements in quality of life and well-being and reduced symptoms of depression. Most quantitative studies were limited by small sample sizes and lack of comparison. All but one study (*n* = 14; 93%) reported qualitative outcomes. The qualitative data showed that the group community was a valued aspect, and participants reported positive outcomes related to health and well-being. In-depth analysis found that participation in SR groups may lead to a renewed sense of personal identity and improved capacity for mentalization.

**Conclusion:**

SR was reported to be a feasible and promising intervention for several groups in different settings. However, available evidence is limited, and research gaps exist. Current findings can serve as a foundation for future studies with larger samples and longer follow-up.

## 1 Introduction

The idea of a relationship between literature and reading and human health dates back to ancient times. In ancient Greece, Apollo was the god of both medicine and poetry. Similarly, Aristotle developed the concept of emotional catharsis among the drama audience of his time, thereby strengthened the connection between literature and personal and psychological growth (Sheppard, 1987). For a long time, books have been used as a treatment for various mental health problems and lifestyle issues, a practice often recognized under the umbrella term bibliotherapy. Bibliotherapy can take many forms, and it can include the use of self-help literature or fiction and poetry, and bibliotherapy can be conducted individually or in group (Canty, 2017). Bibliotherapy using self-help books has shown to be effective to reduce depressive symptoms up to three years after the intervention in adults (Gualano et al., 2017). However, reading books is not equally accessible to all, as it among other things requires reading comprehension and perseverance.

Just after the turn of the millennium the British national charity The Reader in Liverpool developed the group reading concept Shared reading (SR) as an initiative to promote literature and reading (The Reader, 2024). In a SR group meeting, led by a trained facilitator, short stories, novels, and poetry are read out aloud and discussed. No previous preparation is required by the participants – the facilitator hands out the different texts during the session. The participants are thus offered a dialogical interaction in a social context, where they interact with texts written by different authors as well as with their co-participants and the facilitator leading the group sessions. Normally, a SR group consists of 8–12 participants and meets weekly for 1 or 2 h. Although the basic concept is quite clear, there is room for variation and adaptation of the length of the sessions, the session intervals, and number of participants depending on the context.

In a perspectives article published in *The Lancet* 2009, Jane Davis, the founder of The Reader, highlights the uniqueness of participating in an SR group: “The reading-aloud group model offers something live: the sharing of the experience itself, the reading together, and also the immediate discussion of that complex experience in a social community” (Davis, 2009).

The SR concept has, thus, been suggested to facilitate the recognition of thoughts and feelings. This could in turn positively affect participants’ well-being without directly trying to address a specific problem. As a result, SR has in recent years been tried out as an intervention to promote health and well-being in different populations and settings, and beneficial outcomes such as improved quality of life, less pain and better sleep have been reported (Billington et al., 2013; Longden et al., 2016; Billington et al., 2017). In contrast to several other reading interventions directed toward vulnerable groups, the literature is chosen on its value to the human existence in a broad sense for all groups and is not specifically targeting the illness or problem the participants are facing.

Today, many societies are burdened by high prevalence of mental disorders as well as loneliness (GBD 2019 Mental Disorders Collaborators, 2022). The health care systems are keen to find cost-effective interventions beyond pharmacological and psychotherapeutic treatments. SR has several advantages, as SR is scalable, comes with a low cost, and meets several different needs: social, cognitive, cultural, and existential. There is a low threshold to participate in an SR group as it requires no preparation or prior knowledge. Participants are invited and encouraged to reflect on the literature, but there is no pressure to perform or say anything. SR could also serve as a way for people with cognitive impairments or fatigue to get access to literary experiences (Billington et al., 2013; Longden et al., 2016; Andersen, 2022). Libraries constitute an existing infrastructure for conducting SR in terms of facilities, personnel, and material (books).

Other reading interventions aiming to improve mental health and well-being have been studied for a longer time and enough data have been reported to conduct systematic reviews and meta-analysis (Gualano et al., 2017; Wang et al., 2020; Zanal Abidin et al., 2021). However, since the specific SR-concept is a relatively new intervention, the number of publications is limited, and the existing studies have been conducted in various scientific disciplines (psychology, anthropology, literary studies etc.) making it difficult to synthesize the data in a systematic review. Scoping review is a form of review suitable to map the evidence in relatively new areas, where there is a diversity of methods, and an overview is needed to identify research gaps. Therefore, we undertook this scoping review to map the existing SR evidence and identify areas appropriate for further research by addressing the following questions: For whom and in what settings has SR been used? How have the interventions been carried out? What are the outcomes reported? How have outcomes been conceptualized and assessed? What further research is needed to better capture the effects of SR?

## 2 Methods

We followed the PRISMA guidelines for Scoping reviews (Tricco et al., 2018). However, there was no preregistered review protocol for this scoping review.

### 2.1 Eligibility criteria

To be included in the review, papers needed to study SR as an intervention to promote health and/or well-being in adults (age ≥ 18 years) and report original data. Both quantitative, qualitative, and mixed-methods papers could be included. Papers should be written in Danish, English, Norwegian, or Swedish, as these are the languages the authors of this scoping review are proficient in. To be included, the results should be published in peer-reviewed journals between Jan 1, 2005, and Dec 31, 2024. The starting date was chosen in relation to when the SR-concept was introduced.

Studies were excluded if they studied reading interventions other than the SR concept developed by The Reader as this scoping review focuses on this specific method. SR studies not reporting health or well-being outcomes were also excluded.

### 2.2 Search strategy

To identify potentially relevant studies, a search was conducted in LUBsearch, the collective entry point to all the Lund University Libraries’ joint resources. For a full list of indexed databases in LUBsearch, see Table 1. PubMed was also searched. The search was made with the help of university librarians. The final search strings are presented in Table 1.

**Table 1:** Indexed data bases in LUBsearch and the final search strings.

Indexed data bases in LUBsearch^1, 2^
Oxford Competition Law, SAE Mobilus, SveMed+, SwePub, Rock’s Backpages, APA PsycBooks, APA PsycInfo, APA PsycArticles, Idunn.no, Scopus®, Open Textbook Library, Oxford Reference, Encyclopedia of the Bible and its Reception Online, Inspec, GeoRef, GeoRef In Process, Bibliography of Asian Studies, Gale eBooks, ERIC, Atla Religion Database with AtlaSerials, AMED - The Allied and Complementary Medicine Database, Regional Business News, Teacher Reference Center, MLA Directory of Periodicals, MLA International Bibliography, EconLit, Avery Index to Architectural Periodicals, MEDLINE, SAGE Knowledge, Publications New Zealand Metadata, Milne Open Textbooks, AGIS Plus Text / AGIS Index, SocINDEX with Full Text, LGBTQ+ Source, Old Testament Abstracts, Humanities International Complete, Business Source Complete, New Testament Abstracts, Library, Information Science & Technology Abstracts with Full Text, Literary Reference Center, Academic Search Complete, Urban Studies Abstracts, Political Science Complete, Philosopher’s Index, GreenFILE, European Views of the Americas: 1493 to 1750, Arts & Humanities Citation Index, Science Citation Index Expanded, Social Sciences Citation Index, ScienceDirect, Criminal Justice Abstracts with Full Text, Oxford Scholarship Online, Oxford Handbooks Online, arXiv, eBook Collection (EBSCOhost), LexisNexis Academic: Law Reviews, Supplemental Index, Complementary Index, Oxford Art Online, Grove Music Online, Archive of European Integration, Industry Studies Working Papers, Minority Health Archive, Aphasiology Archive, PhilSci Archive, Britannica Online, Directory of Open Access Journals, Persée, HeinOnline, OAPEN Library, British Library EThOS, SSOAR – Social Science Open Access Repository, LUNA Commons, APA PsycTests, BioOne Complete, ePublications, Communication Source, Adam Matthew Digital, Swedish National Bibliography, Oxford Bibliographies, JSTOR Journals, Books at JSTOR, Emerald Insight, SpringerMaterials, OJS vid Lunds Universitet, MathSciNet via EBSCOhost, eScholarship, Oxford Public International Law, IEEE Xplore Digital Library, Art & Architecture Source, SCOAP3, Elgaronline, Springer Nature Journals, Oxford Research Encyclopedias, ACM Full-Text Collection, Wiley Online Reference Works, Archives Unbound, Directory of Open Access Books, Henry Stewart Talks, University Press Scholarship Online, CogPrints, Building Types Online, Cochrane Database of Systematic Reviews, CINAHL Complete, SpringerProtocols, Sustainable Organization Library (SOL), OECD iLibrary, Networked Digital Library of Theses & Dissertations, Cambridge Core Books, BrillOnline Reference Works, OpenDissertations, Very Short Introductions Online (VSI), Routledge Handbooks Online, Library catalogue (LUBcat), Bloomsbury Collections, IMF eLibrary, eBook Subscription Harvard Business Publishing Collection (EBSCOhost), SAGE Research Methods, Economist Historical Archive, CAB eBooks, New Palgrave Dictionary of Economics Online, ProjectMUSE, BDSL (Bibliographie der Deutschen Sprach-und Literaturwissenschaft), Oxford Legal Research Library, Springer Nature eBooks
Search string 1
(“reading therap*” OR “literature therap*” OR “reading as therap*” OR “literature as therap*” OR “literature-based intervention*” OR “reading and mental health” OR “reading for mental health” OR “reading intervention” OR “bibliotherap*” OR “book therapy” OR “therapeutic storytelling” OR “creative arts therapy” OR “poetry therapy” OR “collaborative reading*” OR “social reading*” OR “supportive psychotherapy” OR “mental health problems”) AND “shared reading”
Search string 2
(“reading therap*” OR “literature therap*” OR “reading as therap*” OR “literature as therap*” OR “literature-based intervention*” OR “reading and mental health” OR “reading for mental health” OR “reading intervention” OR “bibliotherap*” OR “book therapy” OR “therapeutic storytelling” OR “creative arts therapy” OR “poetry therapy” OR “collaborative reading*” OR “social reading*” OR “supportive psychotherapy” OR “mental health problems”) AND “shared reading”
Search string 3
“shared reading” NOT (preschool* OR “primary school*” OR infancy OR infant* OR kindergart* OR children OR parental OR classroom OR childhood OR pediatric* OR autis* OR “intellectual disabilit*”)

^1LUBsearch, is the collective entry point to all the Lund University Libraries’ joint resources. 2A separate search was made in PubMed.^

**Figure 1:**
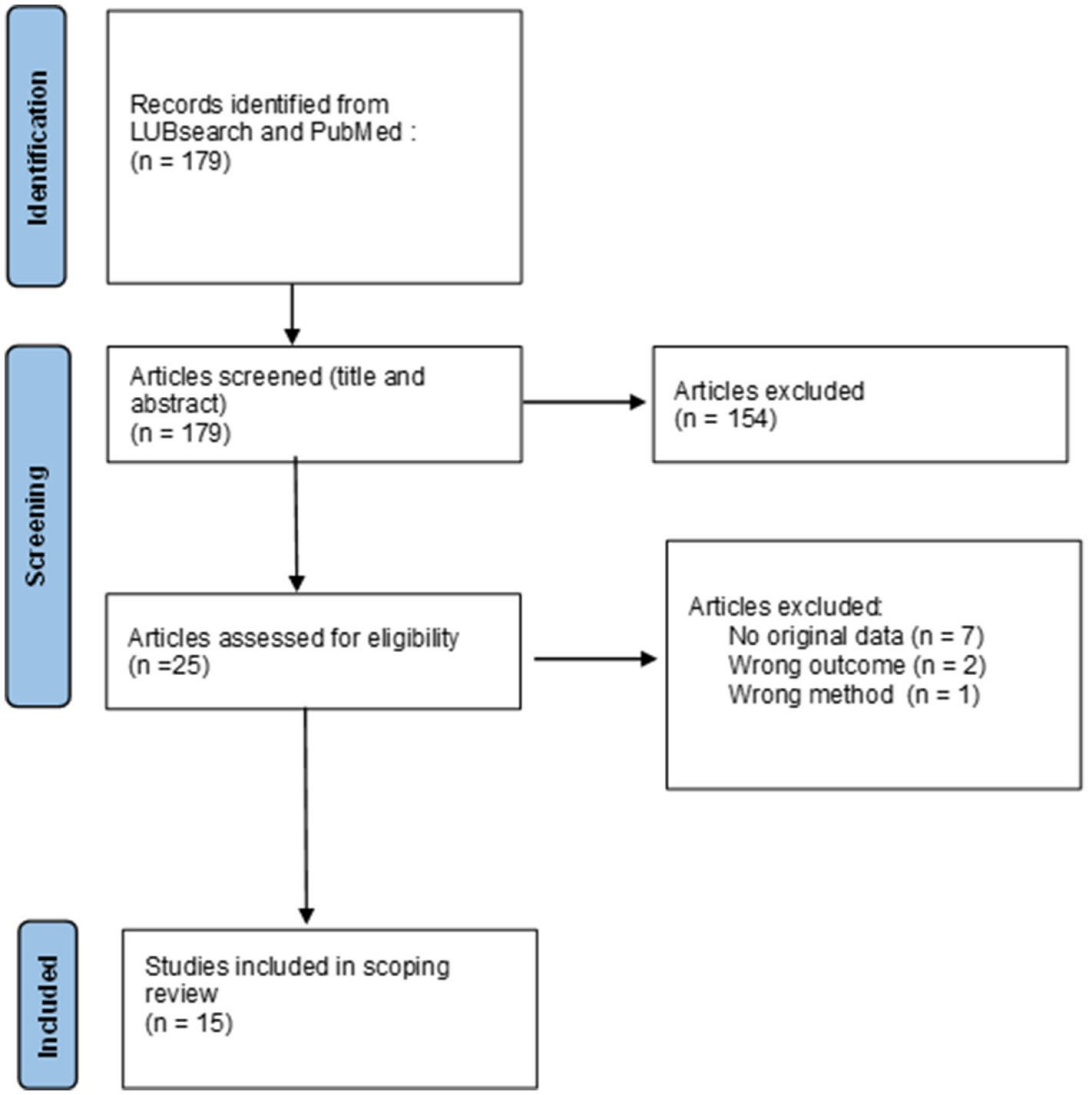
Flow chart of identified, excluded, and included studies for the scoping review.

The number of studies identified in the search is shown in Figure 1. The final search was conducted on Jan 22, 2025.

### 2.3 Selection of sources of evidence

All papers were reviewed (first title and abstract, then full text) by two members of the research team (KJ and TF). Divergent views were resolved through discussion.

### 2.4 Data charting process

A table was created as a charting form, and information from each paper was listed under the headings: country, setting, participants/population, intervention (how SR was delivered, literature used, number of sessions, frequency, closed or open groups etc.), assessment (how was the effect assessed), outcomes (including attrition), limitations, and funding. All co-authors of the present paper independently reviewed the papers (two reviewers for each paper) and added data to the table. One paper was authored by several authors of the present scoping review (Ohlsson et al., 2018), and was therefore deliberately reviewed by two co-authors (MM and JW) not involved in the study by Ohlsson et al.

### 2.5 Synthesis of the results

We have grouped the studies based on the following charting terms: Country, setting, funding, participants, intervention, quantitative and qualitative assessment, quantitative and qualitative outcomes, and reported limitations.

## 3 Results

### 3.1 Selections of sources of evidence

The search yielded 179 papers. Based on title and abstract, 154 papers were excluded. We retrieved 25 studies for full text review, of which ten were excluded. Reasons for exclusion were not containing original data, i.e., commentaries and method papers (*n* = 7), reporting outcomes not relevant, i.e., not focusing on health or well-being (*n* = 2), and using a reading aloud intervention outside the SR concept (*n* = 1). The remaining 15 papers, published between 2007 and 2024, were included in this review. All included papers were written in English. See Figure 1 for a flow chart.

### 3.2 Synthesis of results

The results are presented in Tables 2 and 3 for overview and further elaborated on in text.

**Table 2:** Data extracted from the included studies with information about country, setting, funding, participants, and assessment.

First author and year	1. Country; 2. Setting; 3. Funding	Participants (a. N; b. Target population; c. Age; d. Gender; e. Other demographics)	Intervention (1. Number of session/length of sessions/length of intervention; 2. Literature used)	Quantitative assessment (1. Assessment tools; 2. Assessment points; 3. Other information)	Qualitative assessment (1. Data collection; 2. Method for data analysis)
Andersen (2022)	Norway At a cancer organization and online Horizon Europe, Marie Skłodowska-Curie grant	12 (8 on site, 4 online) People diagnosed with cancer Mean 51 years (range 23–69) Women	16 sessions/90 min/16 weeks Short stories (only a few pages) and poems	None	Participant observation, focus groups, and interviews with the reading leader Open coding, inductive approach. Self-determination theory and the theory Temporarily Expanding the Boundaries of the Self were used as theoretical frameworks.
Billington et al. (2013)	United Kingdom 3 care homes, 2 hospital wards, and 1 day center. The Headley Trust	61 service users and 20 staff members People with dementia Elderly people	One-hour sessions for 3–6 months. Poetry, very short stories, and short extracts from novels. Reading in a much louder voice than usually. Summary of story before discussions.	NPI-Q (staff reported) At baseline and for the care homes every 4 weeks. Longitudinal, quasi-experimental	Semi-structured qualitative interviews with ten staff members who attended SR and/or had extensive knowledge of service users. Thematic analysis
Billington et al. (2016a)	United Kingdom Hospital/pain clinic The University of Liverpool, Department of Culture, Media, and Sport and The Public Engagement Foundation.	6 Patients with chronic pain Not consistently reported Mixed gender	Weekly 2-h sessions for 12 weeks. Fiction (short stories and extracts), poetry.	Pain rating (0–10) with notes about contextual events and medication. BDI, McGill pain index, GHQ, WAS, and MOS. Before, during and after the intervention. Pain rating every 12 h. *N* = 1 time series design.	Initial focus group-interview with participant and project worker. Followed by individual interviews. Thematic analysis
Billington et al. (2016b)	United Kingdom Maximum security prison UK National Personality Disorder Team.	35 Prisoners; many diagnosed with mental health issues 18 to 62 years Women White British; 18% below Adult Literacy Level	Weekly 2-h sessions for 12 months. Poetry, short stories, and extracts from novels.	None	Field observations (7 sessions). Interviews and focus groups with participants, prison staff, project workers/reader leaders. Records kept by the reader leader during group sessions. “Realistic evaluation,” themes were identified from field notes.
Billington et al. (2017)	United Kingdom Pain clinic The British Academy Small Grants Scheme	10 Patients with chronic pain 18 to 75 years 7 women, 3 men White British	22 SR-sessions (SR-only-group). 5 weeks of CBT and then joined the SR group (CBT + SR-group) Literary fiction and poetry.	Pain (0–10) and emotion diary (2 words). PANAS. Twice daily (pain and emotion rating), PANAS after each CBT or SR session	SR and CBT sessions recorded and transcribed. Qualitative interviews with participants. Sessions analyzed with methods which ‘use language as the main point of access to moments of subtle mental change and personal breakthrough, cognitive revaluation, interactive mind’. Findings cross-referenced with participant interviews.
Christiansen and Dalsgård (2021)	Denmark Not reported Not reported	24 in two groups, usually 5 to 6 participants in a session. Mentally vulnerable young people Mid 20 to mid 30. Mixed gender (mostly women) Diverse social background	90 min weekly sessions for 18 months Prose and poetry	None	Ethnographic field work, participant observation, individual and group interviews with 24 participants. Analysis of the emerging atmosphere inspired by Rosenblatt and Gumbrecht.
Dowrick et al. (2012)	United Kingdom GP surgery and a mental health drop-in center Mersey Beat/Liverpool Primary Care Trust	18 at baseline and 8 at follow-up People diagnosed with depression, with ≥1 regular medicine. Majority 35 to 64 years Similar numbers of men and women. White	90 min weekly sessions for 1 year Fiction and poetry	PHQ-9 and a self-reported use of health care services. Before and after the intervention.	Digital recordings of all sessions and diaries completed by the facilitator. 2 members of the research team observed 1 session per month. Conversation analysis and thematic analysis
Hodge et al. (2007)	United Kingdom Libraries, a residential drug rehabilitation unit, and a hostel for homeless men Health Care Education Resources Group Funding, School of Health Sciences, University of Liverpool.	6 groups with 2 to 12 participants. Recovering drug addicts and alcoholics, vulnerably housed men, full-time carers, lone parents, isolated elderly people and facilitators > 18 years	Weekly up to 2 h. Ongoing groups. Novel, short stories, poems, and plays	None	Observations (1 time in 5 groups). Case study (6 times in 1 group). Interviews with stakeholders, participants, and librarians. Thematic analysis
Longden et al. (2015)	United Kingdom The Reader’s head quarter The Arts and Humanities Research Council on Cultural Value	6 persons in SR + 10 volunteers Individuals at risk of or suffering from mental health problems, isolation, or unemployment; volunteers from local community without SR experience. Mean 37.8 years (range 21–70) 11 women and 5 men	6 SR sessions for 90 min + 6 built environment sessions in a cross design. Short stories, novels and poems.	PANAS, DASS-21, DMS, WEMWBS, purpose in life and personal growth subscales from SPWB. Participants also asked to generate two words or phrases that described each session Assessment at baseline and at 6 weeks	Audio and video session recordings. Interviews with participants and mentors. Discourse analysis and ‘realistic evaluation,’ by a multidisciplinary team
Longden et al. (2016)	United Kingdom Four care homes Evaluation commissioned by NHS North West.	31 Care home residents with mild/moderate dementia. Elderly. 16 women, 15 men	One-hour sessions daily for 3 months (2 groups). Weekly session (2 groups) 6–10 participants in each group. Mostly poetry	DEMQOL-Proxy and NPI-Q (staff reported) Assessment at baseline and then every month for 6 months Randomization to intervention or waiting list	None
Ohlsson et al. (2018)	Sweden Not reported Not reported	4 Non-cancer chronic pain patients Not reported Women	90-min, weekly sessions for 8 weeks. The study focuses on one session when a short story was read.	None	Verbatim transcription of video recordings from one SR session. Discursive psychology and Judith Langer’s theory of literary meaning making
Pihl et al. (2024)	Denmark Not reported Not reported	30 Newly, or soon to be, retired seniors 65 to75 years Men Mixed socioeconomic and educational backgrounds	Weekly sessions for 8 weeks Short stories and poems	None	Participant observations. Group interviews with participants. Template analysis
Steenberg et al. (2014)	Denmark Not reported Not reported	8 Well-educated people with a psychiatric diagnosis 30 to 50 years 2 men and 6 women	6 SR sessions for 2 months. Short stories and poems.	Heart rate monitoring Likert-scale assessing motivation, interest, experience, and relatedness to the text. During 4 sessions (heart rate). Immediately after 4 sessions (Likert-scale)	Participant observation at all 6 sessions. 4 sessions audio recorded. Individual interviews with 5 participants and the facilitator 1–2 days after each meeting. Reader-response analysis of the recorded sessions
Tangerås (2022)	United Kingdom Care home Kristiania University College (Norway)	5 to 12 each session (average 7–8) Care home residents with mild to moderate dementia Not reported Mixed gender	Weekly sessions for 12 weeks. Poetry.	None	Field observations and field notes. Theoretical framework borrowed from intersubjective psychotherapy
Watkins et al. (2022)	United Kingdom High secure hospital Mersey Care NHS Foundation Trust	10 at study start, analysis based on 4 regular participants. Patients with experience of psychosis and a history of self-harm. Mean 45.25 years, SD = 6.45 Men All were White British.	Weekly 2-h sessions for 12 months. Short stories and poems	None	39 videos and audio-recorded sessions. Salient sessions were selected for analyze. Psychological discourse analysis

NPI-Q, NeuroPsychiatric Inventory Questionnaire; SR, Shared reading; BDI, Beck Depression Inventory; GHQ, General Health Questionnaire; WAS, Work Adjustment Scale; MOS, Medical Outcomes Study; CBT, Cognitive Behavioral Therapy; PANAS, Positive and Negative Affect Scale; PHQ-9, Patient Health Questionnaire-9; DASS-21, Depression Anxiety and Stress Scale; DMS, Dalgard Mastery Scale; WEMWBS, Warwick-Edinburgh Mental Well-Being Scale; SPWB, Scale of Psychological Well-being; DEMQOL, Dementia Quality of Life SD Standard Deviation.

**Table 3:** Data extracted from the included studies with information about findings and reported limitations.

First author Year	Key quantitative findings	Key qualitative findings	Limitations reported in the paper
Andersen (2022)	Not applicable	Four themes were identified: Open space Disconnecting through connecting Community Resonance and Echos Themes structured in a theoretical model suggesting how SR supports patients with cancer. SR helps balance life and cancer, disconnect from the illness, brings literature back in life, and offers cognitive training. Participants felt useful and valuable to other participants.	Small sample size. No triangulation with audio recordings from the sessions.
Billington et al. (2013)	Lowered NPI-Q scores compared to baseline, i.e., SR may positively influence the behavioral symptoms of dementia.	SR helped trigger memories and possibly contributed to a renewed sense of personal identity. SR influenced quality of life and well-being. Participants enjoyed the sessions. Listening, memory, and attention was enhanced.	Pilot study with limited sample size. No data on reading habits prior to the dementia. No control group. Not possible to conclude that SR caused the positive effects reported.
Billington et al. (2016a)	Some positive changes in reported pain and psychological well-being. This was consistent with participants’ accounts.	Literature regarded as an essential component. Participants appreciated the use of non-pain-related literature and diversity of texts. More challenging texts were preferred leading to absorbed concentration. Group community important and valued. The SR countered the negative effects of pain on mood.	Constantly changing experiences hard to capture. Larger sample sizes are needed. No control group.
Billington et al. (2016b)	Not applicable	Attendance rates good. SR elicited memories. The literature used as a connection to a continuing life outside prison. SR facilitated mentalization, i.e., understanding own and others’ thoughts and feelings. Improved social, emotional/psychological, and educational well-being.	Data collection constrained by the custodial setting. Brief and sporadic research visits Lack of quantitative assessment. Lack of control group.
Billington et al. (2017)	Lower pain rating and higher emotion rating in days following SR. The same positive effects not seen after CBT (*n* = 3). Positive emotions more prevalent than negative directly following sessions (both in SR and CBT). Greater diversity and intensity among the two chosen words after SR.	Different focus in CBT (health conditions, personal experience, and difficulties) and SR (diversity of subjects prompted by literary text, language signaling perspectivization and change). Participant reported feeling more relaxed and sleeping better after SR. Intimacy, diversity, and collaboration in the group valued.	Small sample size. No comparison with interventions with other kind of shared material.
Christiansen and Dalsgård (2021)	Not applicable	During SR participants briefly tuned into a collective atmosphere of presence (a momentary transformation) arising from the collective engagement in the literary text.	Difficult to separate the effect of SR from other factors in the participant’s lives.
Dowrick et al. (2012)	Significantly reduced symptoms of depression at follow-up (PHQ-9). No reduction in the use of health services at follow-up.	Three core components identified as potentially important for therapeutic efficacy: SR of literary texts skilled facilitation social group processes.	Small sample size. Lack of control group. Lack of standardized interviews to diagnose depression. High attrition. No information regarding other ongoing treatments.
Hodge et al. (2007)	Not applicable	Participants appreciate the social function of the group and the diversity of group members. Supporting ongoing group processes in therapeutic groups. Increased enthusiasm for literature and reading. Reading groups have significant potential to increase well-being.	Not discussed.
Longden et al. (2015)	Both activities associated with higher levels of positive affect than negative affect. The activities promoted different aspects of well-being. Purpose in life improved after SR but not after BE.	Five intrinsic values of SR were identified: Liveness Creative inarticulacy Emotional Personal The group SR offered richer emotional content compared with BE. BE provided opportunities to look forward positively beyond ‘the self’ and into the community, SR was more engaged with the introspective and the past.	Small sample size. Underpowered study. Low levels of affective symptoms at baseline making improvements less likely.
Longden et al. (2016)	Positive and sustained effects (3 months) on quality of life after SR.	Not applicable	Small sample Lack of control for confounding variables. Low levels of baseline symptoms prevented analyses on whether the intervention impacted on the clinical signs of dementia.
Ohlsson et al. (2018)	Not applicable	Group members have been able to perform acts of mentalization, i.e., understanding mental acts of literary characters as well as fellow group members.	Future studies are needed, and perspectives need to be broadened and integrated.
Pihl et al. (2024)	Not applicable	Two dominant themes were identified: the articulation effect of literary texts – the SR participants understood better their own lives as well as other people’s viewpoints SR facilitated identification and social connectivity.	Lack of a control group. However, the aim was not to compare.
Steenberg et al. (2014)	Inverse relation between feeling related to the text and heart synchronization	The text is a significant agent of the shared/subjective reading experience, and the facilitation is important for making the text an agent. Synchronization (instead of reading being a process of differentiation and synchronization) is an effect of the text no longer being an active agent.	Not discussed.
Tangerås (2022)	Not applicable	Description of 8 different ‘now moments’ that became ‘moments of meeting’ during the different SR sessions	Long-term changes in participants not analyzed.
Watkins et al. (2022)	Not applicable	Archetypes of interactional achievement over the year with the SR intervention were identified via certain rhetorical strategies. These improvements were illustrated by the four regular participants individually (although not exclusive to the unique participant): one participant showed a broadening capacity to alternative interpretations of events one showed increased assertiveness one showed decreased avoidance one showed heightened engagement	Not possible to separate the effect of SR from the effect of other ongoing interventions such as psychological therapy and medication. The sample consisted of only men, and it is not known if the findings are transferable.

SR, Shared reading; NPI-Q, NeuroPsychiatric Inventory Questionnaire; CBT, Cognitive Behavioral Therapy; PHQ-9, Patient Health Questionnaire-9; BE, Built Environment.

#### 3.2.1 Countries

So far, SR as an intervention to improve mental health and well-being has been studied mainly in the UK (ten studies; 67%) (Hodge et al., 2007; Dowrick et al., 2012; Billington et al., 2013; Longden et al., 2015; Billington et al., 2016a; Billington et al., 2016b; Longden et al., 2016; Billington et al., 2017; Watkins et al., 2022; Tangerås, 2022), but also in the Scandinavian countries (33%): Denmark (three studies) (Steenberg et al., 2014; Christiansen and Dalsgård, 2021; Pihl et al., 2024), Norway (one study) (Andersen, 2022), and Sweden (one study) (Ohlsson et al., 2018).

#### 3.2.2 Populations and settings

Targeted groups have generally been different vulnerable populations, such as people with psychiatric disorders (Dowrick et al., 2012; Steenberg et al., 2014; Watkins et al., 2022), patients with chronic pain (Billington et al., 2016a; Billington et al., 2017; Ohlsson et al., 2018), patients with cancer (Andersen, 2022), elderly with dementia (Billington et al., 2013; Longden et al., 2016; Tangerås, 2022), recovering drug addicts (Hodge et al., 2007), and prisoners (Billington et al., 2016b), but SR has also been used to improve well-being in newly retired seniors (Pihl et al., 2024) (Table 2).

The SR sessions have been conducted in a variety of naturalistic settings such as care homes (Billington et al., 2013; Longden et al., 2016; Tangerås, 2022), clinics/hospitals (Dowrick et al., 2012; Billington et al., 2013; Billington et al., 2016a; Billington et al., 2017; Watkins et al., 2022), prisons (Billington et al., 2016b), and libraries (Hodge et al., 2007) (Table 2). Dowrick et al. (2012) reported that the setting had an important influence on SR as it was easier to engage participants at the mental health drop-in center compared to at a GP surgery, where the participants initially regarded the literature as something “prescribed.” In a Norwegian study published in 2022, some of the participants had taken part in SR online (Andersen, 2022).

Most studies included in this review have studied small samples, and five studies had ten or fewer participants (Steenberg et al., 2014; Billington et al., 2016a; Billington et al., 2017; Ohlsson et al., 2018; Watkins et al., 2022). The largest study included 61 participants (Billington et al., 2013). Some studies have purposely studied participants with a specific gender, such as female prisoners (Billington et al., 2016b) or newly retired men (Pihl et al., 2024). Other SR groups were open to all genders but attracted only women (Ohlsson et al., 2018; Andersen, 2022). Participants’ age was not systematically reported in all studies and ranged from 18 to “elderly” (Table 2).

#### 3.2.3 Funding

Funding was reported in 9 out of 15 studies. The studies were primarily funded by health care authorities, research councils, and universities (Table 2).

#### 3.2.4 Intervention

The interventions varied between 6 and 60 sessions, distributed over 2 to 18 months (Table 2). When reported, length of sessions ranged between 1 and 2 h, and were commonly delivered weekly. However, in a study with participants with dementia, two out of four groups were offered daily instead of weekly sessions (Longden et al., 2016).

In most studies, fiction and poetry were used in the reading sessions. In some cases, adaptations were made in relation to the targeted group, such as shorter texts used for people with dementia (Billington et al., 2013) and people with cancer-related fatigue (Andersen, 2022). Other reported adaptions were the facilitator reading in a much louder voice than usual and summarizing the story before discussion when the target group was people with dementia (Billington et al., 2013; Longden et al., 2016).

#### 3.2.5 Study design and data collection

Seven of the 15 studies (47%) collected some form of quantitative data (Dowrick et al., 2012; Billington et al., 2013; Steenberg et al., 2014; Longden et al., 2015; Billington et al., 2016a; Longden et al., 2016; Billington et al., 2017). All studies collecting quantitative data had a prospective design with repeated assessment. In all studies with post-treatment assessment, this assessment was done in close proximity to the end of the intervention, but no study reported long-term follow-up. A few studies used strategies to improve reliability such as double pre-assessment (Billington et al., 2013), and one study had a randomized controlled design (Longden et al., 2016). Quantitative data were collected using questionnaires, pain rating, and heart rate monitoring (Table 2).

All but one study (Longden et al., 2016) collected qualitative data (93%). The data were collected with participant observations, recordings of sessions, diaries, focus groups, and interviews (Table 2). Both participants and facilitators were interviewed. The method for analyzing qualitative data varied between the studies (Table 2). Thematic analysis was the most frequently reported method (Hodge et al., 2007; Dowrick et al., 2012; Billington et al., 2013; Billington et al., 2016a).

#### 3.2.6 Outcomes

##### 3.2.6.1 Quantitative studies

Six studies reported positive effects of SR (Table 3). Positive changes were reported for depressive symptoms (Dowrick et al., 2012), dementia symptoms (Billington et al., 2013), pain (Billington et al., 2016a; Billington et al., 2017), psychological well-being and quality of life (Longden et al., 2015; Billington et al., 2016a; Longden et al., 2016), and positive affect (Longden et al., 2015; Billington et al., 2017). One study reported no reduction in the use of health services after the intervention (Dowrick et al., 2012). No study reported adverse effects of the intervention.

##### 3.2.6.2 Qualitative studies

The qualitative observations had different focuses. Several studies reported on SR’s social function and the participants talked about the group community being helpful and valued (Hodge et al., 2007; Billington et al., 2016a; Billington et al., 2017; Andersen, 2022). Several studies reported improvements of different mental aspects such as memory, attention, well-being, quality of life, mood, less focus on negative effects of pain, and better sleep (Billington et al., 2013; Billington et al., 2016a; Billington et al., 2016b; Billington et al., 2017). Also, more fundamental mental effects were reported, such as a renewed sense of personal identity and improved capacity for mentalization (Billington et al., 2013; Billington et al., 2016b; Ohlsson et al., 2018).

In studies addressing the literary components of the SR intervention, positive educational effects were reported, as well as increased enthusiasm for literature and reading and a richer reading experience gained through SR (Hodge et al., 2007; Andersen, 2022). Diversity and complexity of the texts were appreciated as well as the texts not being related to the participants’ specific problems (Billington et al., 2016a). Beyond this, the literature, as well as the related discussions, were described as promoting a change of perspectives and a sense of connectedness to the outer world (Billington et al., 2016b; Andersen, 2022).

Two studies reported on the importance of the group facilitator, indicating that the facilitator is a key component of the SR intervention (Dowrick et al., 2012; Steenberg et al., 2014).

#### 3.2.7 Reported limitations

Limitations were reported in 12 out of 15 studies. Mentioned limitations were small sample sizes, short follow-up, no assessment at baseline or few symptoms at baseline, no control group, attrition inflicting bias, and no information about other parallel interventions (Table 3).

## 4 Discussion

For this scoping review, we found 15 papers – published between 2007 and 2024 – reporting data from SR interventions to improve health and well-being in adults. The use of SR in various settings with different groups indicates a willingness to explore culture-based health interventions and is in line with the general upswing of arts in health over the past decades (Fancourt, 2017). In line with other forms of reading interventions to improve mental health and well-being (Canty, 2017; Gualano et al., 2017; Wang et al., 2020), a majority of the SR studies showed promising results, sparking the interest in further exploring SR as an intervention for various groups.

A strength of the reviewed studies is that all were conducted in naturalistic settings and had few exclusion criteria. This indicates a high ecological validity and feasibility. Targeted groups in the reviewed studies were predominantly vulnerable populations of different kinds, showing that SR is feasible for a range of different groups.

Overall, the analyzed studies showed high fidelity to the SR concept, such as having a reading group with a facilitator, reading aloud, and discussing poetry and short stories for 1 or 2 h. However, a great variation in the number of sessions, number of participants, and length of the interventions makes it difficult to assess more precisely the frequency of SR necessary to be effective. It is not known if a single, deeply profound SR session could elicit significant change. However, in the study by Watkins et al. (2022), analyzing the effect of SR on the participants’ discourse, the changes were discernible from around 6 months. As a result, it is difficult to estimate the resources necessary to deliver an effective SR intervention. Still, SR and other reading interventions (Gualano et al., 2017), in comparison with many other types of health interventions (nature-based interventions, art therapy, animal assisted therapy, virtual reality therapy etc.) may be a potentially cost-effective and scalable intervention.

The analyzed SR interventions were carried out in the UK and the Scandinavian countries. This may be due to the selection criteria. Only studies published in English, Danish, Norwegian, and Swedish could be included in this review, although only studies in English were found. There may be papers trying out SR as an intervention to improve health and well-being in adults published in other languages that were not included in this scoping review. The fact that a majority of the studies were conducted in the UK is, however, in line with SR being created and developed at the University of Liverpool. Several of the reviewed studies with data from the UK have been conducted by researchers connected to the founding SR organization (Hodge et al., 2007; Billington et al., 2013; Longden et al., 2015). Likewise, most Scandinavian studies have been carried out by researchers who have introduced SR – or are collaborating with the introducers – in the respective countries (Ohlsson et al., 2018; Christiansen and Dalsgård, 2021; Tangerås, 2022). Researchers trying out interventions they have developed or interventions they are highly invested in can be expected to be more dedicated, adherent, and enthusiastic than other practitioners, which may influence the outcomes. Such a dedication effect is something else than the intrinsic effect of the intervention per se, and in the future, SR needs to be evaluated by researchers less invested in the concept (Durlak and DuPre, 2008). In relation to funding, the studies included in this review were funded by health care authorities, research councils, and universities. We have not found any specific commercial interests that would benefit from the SR concept being implemented or promoted.

The existing studies have laid the ground for upcoming studies, by showing feasibility for different groups in different settings and promising effects on mental health and well-being (Dowrick et al., 2012; Billington et al., 2016a; Longden et al., 2016; Billington et al., 2017) as well as more fundamental mental effects such as a renewed sense of personal identity and improved capacity for mentalization (Billington et al., 2013; Billington et al., 2016b; Ohlsson et al., 2018). However, future studies need more scientific rigorousness, and the findings need to be replicated, and other forms of reading interventions have more solid evidence at the moment (Gualano et al., 2017). Most studies included in this review were limited by small sample sizes and lacked a control group. Attrition was high in some studies (e. g. 56% in Dowrick et al., 2012 and 60% in Watkins et al., 2022) or not reported. Post-intervention assessments were mostly done in connection to the end of intervention, and little is known about the long-term effects of participating in an SR group. However, Longden et al. (2016) reported sustained effects in people with dementia 3 months after the end of intervention. The effectiveness of different intensities and length of SR should also be compared. Further, SR interventions also need to be compared with other types of social interventions where people meet and interact around a common task, which so far has only been done by Longden et al. (2015). Effective therapeutic interventions potentially have side effects such as deterioration of symptoms, or onset of new symptoms (Rozental et al., 2016), and future studies also need to address potential adverse outcomes.

Other reading interventions have successfully been conducted online (Hoover et al., 2023). In the reviewed studies only one used SR digitally (Andersen, 2022), and it is not known whether SR has the same effect on mental health and well-being when delivered online compared to in-person. In a Swedish study on an online SR group during the COVID pandemic, the participants did not perceive the online format as negatively affecting the group cohesion, instead highlighting that it allowed for more focused attention on the text and the discussion (Gustafsson et al., 2023). However, this group was not aiming at improving the participants’ health and well-being. Some groups could especially benefit from participating digitally, e.g., if participants in a targeted group are geographically spread or if showing up in real life is experienced as too exhausting. Future studies could compare the outcomes between SR delivered in-person versus digitally, potentially in a randomized controlled trial.

So far, there is a lack of common terminology and a methodological consensus within the SR studies. This makes comparison between studies difficult. Consistency in qualitative and quantitative methods over several studies to compare results would be preferable. As an example, the same questionnaires could be used between studies to assess mental health and well-being before and after the SR group. Longden et al. (2015) have suggested that it is necessary to use specific and sensitive questionnaires, since SR may affect specific aspects of well-being such as purpose in life. There is also a need for replicated studies on specific target groups. Only one of the reviewed studies had used a biological marker to study the effect on the participants (Steenberg et al., 2014), and future studies could potentially study SR’s effect on brain connectivity (Berns et al., 2013). The qualitative studies on SR have been conducted in different academic disciplines and on different levels, using different methods for collecting and analyzing the data. A strength is that different perspectives have been explored, but different research practices and traditions make it difficult to evaluate the studies against the same quality criteria and perform a qualitative synthesis of the results.

The specific literary components of the SR intervention were addressed in some studies, but not on a content level so much as regarding the format: in a study with patients diagnosed with cancer the chosen stories were short due to fatigue being a common symptom among the patients (Andersen, 2022). In a similar manner, poetry has been the preferred choice in interventions for elderly people with mild to moderate dementia (Billington et al., 2013; Longden et al., 2016). However, most studies lack a thorough discussion of the literary texts and the specific way they challenge and affect the participants. In some studies, all literature used is listed, e.g., Billington et al. (2017), whereas other studies do not mention the titles of the texts used. In such studies, the literature is an unknown ‘stimulus’ the participants are responding to rather than a central agent.

As acknowledged above, using literature to improve mental health and well-being is not just a recent phenomenon (Canty, 2017), and SR has not been evaluated against other reading interventions. So far, other forms of bibliotherapy have more scientific evidence, with data from several randomized controlled trials and published systematic reviews (Gualano et al., 2017; Zanal Abidin et al., 2021) and also meta-analysis (Wang et al., 2020). In more traditional bibliotherapy the literary content is often specifically chosen for the participants as a therapeutic tool and self-help books can be used (Canty, 2017; Jack and Ronan, 2008). Participants with different health issues or in certain circumstances may find targeted SR groups especially valuable, even if the literature read and discussed during SR is not focusing on the specific illness or life circumstances. It may be valuable to be able to meet with people in the same situation, without having to interact by talking about the common problem (Andersen, 2022). Sharing and discussing poetry and fiction with others can be an appreciated mental challenge and improve self-esteem beyond what a support group can accomplish (Billington et al., 2016a).

It is not only the chosen and read aloud fictional texts that may affect the result of the intervention, but also the facilitator. A few studies explored the importance of the facilitator in the SR concept, and it is not known if a skilled reader leader gets a significantly better result than a less skilled one, and what qualities of the facilitator that are most important. Thus, the facilitator’s role needs to be addressed in future studies, analyzing the actions taken by the facilitator in relation to the group and the texts. Both the literature chosen and the role of the facilitator can be described as mechanisms within the SR group which produce the potential effects. Another such mechanism to explore in future studies is the interaction between the participants necessary to create an effect, as SR may offer unique opportunities to practice perspective taking and mentalization (Gustafsson et al., 2023).

### 4.1 Limitations

This scoping review has some limitations. With the focus on SR only, we have not included other read aloud interventions for adults aiming at improving mental health and well-being not labeled as SR, e.g., (Blundell and Poole, 2023). Including only papers from peer-reviewed journals may have excluded data reported in other types of publications, such as books and dissertations. There might be publications in other languages that were not included in this scoping review. A strength is that we followed the PRISMA guidelines for Scoping reviews as far as possible, which contributed to the work being carried out in a systematic way.

## 5 Conclusion

This scoping review gives an updated overview of where the SR research focusing on health and well-being stands today and can serve as a foundation when future studies are designed. SR so far appears to be feasible and appreciated by participants and facilitators, and is potentially an effective and cost-effective intervention, filling an important societal need. However, this scoping review shows that more research is necessary before implementation in clinical care. At present, there is a lack of large, controlled studies comparing SR to other potential interventions to the targeted groups. Also, long-term studies showing lasting benefits from SR are lacking. A key future research question, beyond the scope of this review, is also if SR could be used to prevent mental illness.
